# De‐Escalation of Disease‐Modifying Therapy in Multiple Sclerosis—A Danish Nationwide Cohort Study

**DOI:** 10.1111/ene.70042

**Published:** 2025-02-02

**Authors:** Frederik Elberling, Mie Reith Mahler, Luigi Pontieri, Finn Sellebjerg, Melinda Magyari, for the DMSG Study Group

**Affiliations:** ^1^ The Danish Multiple Sclerosis Registry, Danish Multiple Sclerosis Center, Department of Neurology Copenhagen University Hospital – Rigshospitalet Glostrup Denmark; ^2^ Department of Clinical Medicine University of Copenhagen Copenhagen Denmark

**Keywords:** age, DMT, high‐efficacy therapy, moderate‐efficacy therapy, MS, risk benefit, treatment strategy, treatment switch

## Abstract

**Background and Objective:**

High‐efficacy (HE) disease‐modifying therapies (DMT) are increasingly used to treat multiple sclerosis (MS). Concerns arise when considering the decreasing efficacy and increasing risk of adverse events in aging patients. We aimed to describe disease activity and treatment trajectories in patients with MS who de‐escalated from an HE DMT to an moderate‐efficacy (ME) DMT.

**Methods:**

We performed a cohort study based on data from the Danish Multiple Sclerosis Registry (DMSR) including patients with relapsing–remitting MS (RRMS), who switched from an HE DMT to an ME DMT as defined by Danish authorities. We included patients from October 2007 to July 2023. Median follow‐up time was 0.8 years (IQR 0.3–2.5).

**Results:**

In total 333 patients (76.0% females, mean age: 45.1 years) de‐escalated for various reasons. Most patients de‐escalated from natalizumab or fingolimod (43.8% and 42.0%, respectively) to dimethyl fumarate (47.5%). At 2 years after de‐escalation, the cumulative risk of relapse was 38% (95% CI 31–44) and 53% (95% CI 46–60) for inflammatory disease activity (relapses and/or radiological disease activity). Age (HR 0.96, 95% CI 0.94–0.98) and inflammatory disease activity prior to de‐escalation (HR 2.05, 95% CI 1.45–2.91) were associated with inflammatory disease activity post de‐escalation.

**Discussion:**

De‐escalation from primarily natalizumab and fingolimod did not effectively ensure disease stability in this cohort. Younger age and inflammatory disease activity prior to de‐escalation were risk factors for inflammatory disease activity post de‐escalation, which can help guide future studies on de‐escalation.

## 1 Introduction

Multiple sclerosis (MS) is a chronic, immune‐mediated neurological disorder characterized by inflammation, demyelination, and axonal loss [1]. Over the past few decades, the therapeutic landscape for MS has broadened with the emergence of several disease‐modifying therapies (DMTs) that vary in their mechanisms of action, efficacy, and safety profile. Initially, the treatment approach for MS was based on starting with relatively safer but less efficacious agents, also termed moderately effective (ME) DMTs, and escalating to more potent therapies, or highly effective (HE) DMTs, as the disease progressed or in response to breakthrough disease activity, which is known as an escalation treatment strategy [2].

However, as evidence from real‐world studies has accumulated, a paradigm shift has emerged [3]. A “hit hard and early” strategy has been advocated, where HE DMTs are initiated in the early stages of the disease to prevent potential long‐term disability [4, 5]. While this approach has its merits, concerns arise when considering the long‐term side effects and safety profiles of HE DMTs, especially in aging patients and patients with low to moderate disease activity who might have achieved similar outcomes on an ME DMT [6, 7].

As individuals age, a decrease in inflammatory activity is observed, whereas the gradual progression of disability becomes increasingly prominent [8]. Data suggest that the effectiveness of DMTs diminishes with increasing age and that HE DMTs and ME DMTs on average might be equally effective in terms of disability progression in patients older than 40.5 years [9, 10, 11]. Concurrently, the risk of infections increases due to age‐related changes to the immune system, known as immunosenescence, as well as with higher disability levels [7, 12, 13].

De‐escalation involves different strategies such as extended or reduced dosing, switching from HE DMT to ME DMT, or treatment discontinuation [7]. Several studies have focused on treatment discontinuation and extended interval dosing, however, studies that investigate a switch from an HE DMT to an ME DMT are limited in number and have mostly focused on natalizumab [7, 14].

This study aimed to investigate disease activity and treatment trajectories in patients with MS who de‐escalated from a broad range of approved HE DMTs to ME DMTs for various reasons. Further, we sought to identify factors associated with inflammatory disease activity following de‐escalation.

## 2 Materials and Methods

### 2.1 Data Source

Thirteen MS clinics in public hospitals in Denmark exclusively provide clinical management and treatment with DMTs for patients with MS. The DMSR was established in 1956 and continuously collects data on various pre‐ and on‐treatment parameters from all patients [15]. For patients to receive DMTs, treating neurologists must enter information on patients with MS as part of routine follow‐up. Expanded Disability Status Scale (EDSS) scores, relapses, yearly MRI scans, and treatment changes are documented with corresponding dates.

### 2.2 Study Population

This nationwide cohort study included all patients with relapsing–remitting MS (RRMS) who switched from an HE DMT to an ME DMT between October 2007 and July 2023. Therapies were defined as HE DMTs or ME DMTs based on the Danish Medicines Council's definitions [16]. HE DMTs included ofatumumab, fingolimod, alemtuzumab, rituximab, cladribine, ocrelizumab, natalizumab, daclizumab, siponimod, and ozanimod. ME DMTs comprised teriflunomide, interferon‐β, glatiramer acetate, and dimethyl fumarate. We defined de‐escalation as a switch from a HE DMT to a ME DMT.

We excluded patients stopping HE DMT due to pregnancy, patients with less than 6 months on HE DMT (either one or several consecutive HE DMT treatments totaling > 6 months were required), and those with a gap exceeding 1 year between discontinuing an HE DMT and starting an ME DMT. Patients were followed from the date of ME DMT initiation (baseline) until death, emigration, discontinuing the ME DMT, or July 6, 2023 (end of follow‐up), whichever occurred first.

### 2.3 Outcomes and Covariates

For time‐to‐event analyses, we defined our outcome as relapses in one statistical model, and disease activity (defined as the earliest occurrence of either a relapse, a new or enlarging T2 lesion, or a gadolinium‐enhancing lesion on MRI) in another statistical model. We included the following covariates that were measured at baseline: age, sex, disease duration (since MS onset), number of previous treatments, last available EDSS score before baseline, EDSS increase during the last 6 months of HE DMT, disease activity during the last 6 months of HE DMT (relapses, gadolinium‐enhancing lesions, new or enlarging lesions), and duration of all previous HE DMT combined. EDSS increase was defined as an increase in the EDSS score of 1.5 if the baseline EDSS score was 0, 1 if it was between 1 and 5, or 0.5 if it was above 5.

At the end of follow‐up, we also assessed treatment trajectories post de‐escalation. Patients were categorized into four groups according to the first treatment switch following de‐escalation, if any: re‐escalation (re‐starting an HE DMT), continuation (the same ME DMT until the end of follow‐up), lateral switch (switch to another ME DMT), and discontinuation (if no other DMT was initiated within a year). We then analyzed the age distribution at de‐escalation among the four groups.

### 2.4 Statistical Analysis

Continuous variables were summarized using mean and standard deviation (SD) or median and interquartile range (IQR) as appropriate. Categorical variables were summarized using frequencies and proportions. We included information on missing data in relevant categories.

We estimated the cumulative proportion of patients experiencing relapse or disease activity 2 years after de‐escalation and visualized the proportion of patients without the event with Kaplan–Meier curves. We used two separate multivariable Cox models to estimate the association between baseline clinical and demographic characteristics and the hazard of relapse or disease activity after de‐escalation. We included age (in years), disease duration (in years), number of previous treatments, and EDSS score as continuous covariates. As categorical covariates, we included sex (female or male), EDSS increase during the last 6 months of HE DMT (yes or no), disease activity during the last 6 months of HE DMT (yes or no), and combined duration of all previous HE DMT (below or above 1 year).

We used separate Welch two‐sample *t*‐tests to assess differences in the mean age at initiation of ME DMT among the four post de‐escalation treatment groups (re‐escalation, continuation, lateral switch, and discontinuation). We used the false discovery rate (FDR) method to correct for multiple testing.

A two‐sided *p*‐value of < 0.05 was considered statistically significant. We conducted all statistical analyses using R version 4.2.0 [17] and R packages (“moments” and “survival”) [18, 19].

### 2.5 Standard Protocol Approvals, Registrations, and Patient Consents

In Denmark, register‐based studies using anonymized data do not require informed consent or ethical approval. In compliance with the General Data Protection Regulation, we have modified data cells containing less than three individuals or ones that allow cross‐cell calculations.

## 3 Results

### 3.1 Study Population

**FIGURE 1:**
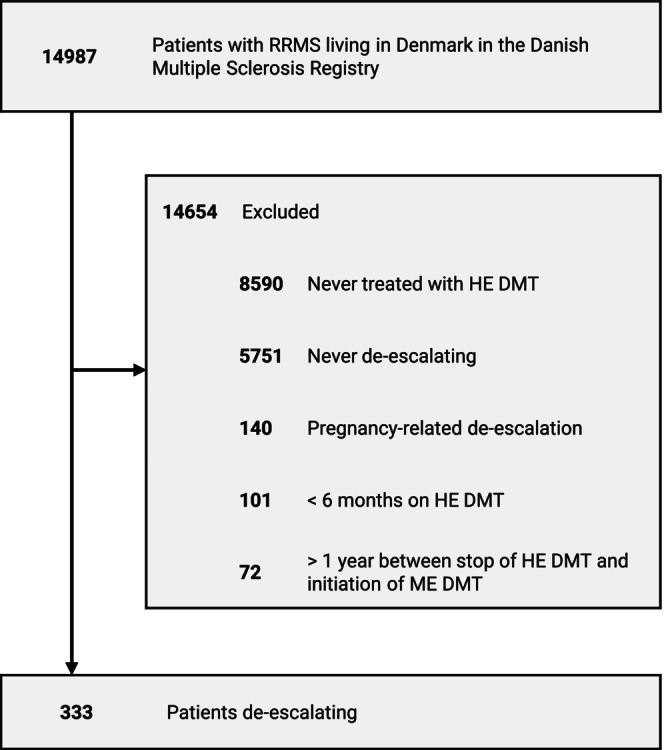
Flowchart of the inclusion process. HE DMT, high‐efficacy disease modifying therapy; ME DMT, moderate‐efficacy disease modifying therapy; RRMS, relapsing–remitting multiple sclerosis.

Figure 1 shows a flowchart of the inclusion process. Of the 333 patients who de‐escalated from an HE DMT to an ME DMT, 253 (76.0%) were female. At baseline, the mean age was 45.1 years (SD 10.1) and patients had a median EDSS score of 3.0 (IQR 2.0–4.5). The mean age at clinical onset was 32.1 years (SD 8.9), with the first HE DMT initiated at a mean age of 41.3 years (SD 9.5) and the last HE DMT prior to de‐escalation initiated at a mean age of 42.7 years (SD 9.4). The median treatment duration of the last HE DMT prior to de‐escalation was 2.8 years (IQR 1.6–5.9).

Most patients de‐escalated from natalizumab or fingolimod (43.8% and 42.0%, respectively), while dimethyl fumarate was the most common ME DMT patients de‐escalated to (47.5%). The main reasons for discontinuing the last HE DMT prior to de‐escalation were adverse events (41.4%), followed by John Cunningham (JC) virus antibody positivity (22.5%), disease activity (11.1%), and development of antidrug antibodies (8.1%). The median time between discontinuing the HE DMT and baseline was 59 days (IQR 26–125) (Table 1).

**TABLE 1:** Demographic and clinical characteristics at baseline.

	Patients de‐escalating treatment (*n* = 333)
Sex, *n* (%)	
Females	253 (76.0)
Males	80 (24.0)
Age at MS clinical onset, mean (SD), years	32.1 (8.9)
Age at MS diagnosis, mean (SD), years	34.6 (9.2)
Age at initiation of first HE DMT, mean (SD), years	41.3 (9.5)
Age at initiation of last HE DMT, mean (SD), years	42.7 (9.4)
Duration of last HE DMT, median (IQR), years	2.8 (1.6–5.9)
Disease activity on last HE DMT,^a^ *n* (%)	89 (26.7)
Duration between last HE DMT discontinuation and ME DMT initiation, median (IQR), days	59 (26–125)
Age at initiation of ME DMT, mean (SD), years	45.1 (10.1)
Last EDSS prior to initiation of ME DMT, median (IQR)	3 (2–4.5)
Missing, *n* (%)	2 (0.6)
Last HE DMT, *n* (%)	
Natalizumab	146 (43.8)
Fingolimod	140 (42.0)
Alemtuzumab	18 (5.4)
Ocrelizumab	11 (3.3)
Rituximab	6 (1.8)
Daclizumab	5 (1.5)
Cladribine	4 (1.2)
Ofatumumab	3 (0.9)
Reasons for discontinuation of last HE DMT, *n* (%)	
Adverse events	138 (41.4)
JCV antibody positivity	75 (22.5)
Disease activity^b^	37 (11.1)
Antidrug antibodies	27 (8.1)
Contra indication	20 (6.0)
Other not specified reason	16 (4.8)
Patient's decision	7 (2.1)
Progression	3 (0.9)
Practical issues	≤ 3^c^
Lack of patient compliance	≤ 3^c^
Stable condition	≤ 3^c^
Missing	3 (0.9)

Abbreviations: DMT, disease modifying therapy; EDSS, Expanded Disability Status Scale; HE, high efficacy; IQR, inter quartile range; JCV, John Cunningham virus; ME, moderate efficacy; MS, multiple sclerosis; SD, standard deviation.

^a^ Disease activity defined as relapses, new/enlarging T2 lesions, or gadolinium‐enhancing lesions on MRI within 6 months prior to discontinuing HE DMT.

^b^ Reason for stopping HE DMT reported as either due to disease activity, relapse, and/or MRI activity.

^c^ Masked to avoid individually identifiable information and to comply with the General Data Protection Regulation (GDPR).

### 3.2 Disease Activity and Disability Accumulation Following De‐Escalation

**FIGURE 2:**
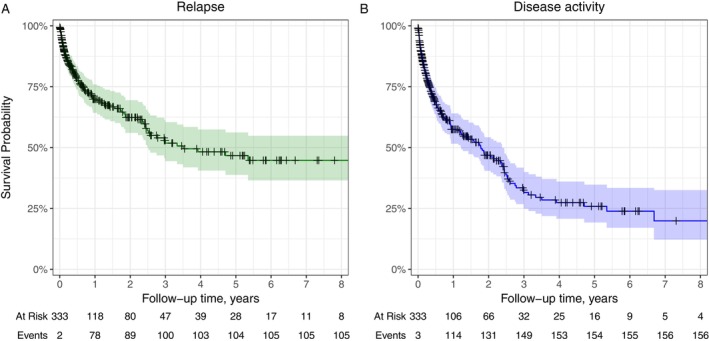
Probability of not experiencing a relapse or disease activity following de‐escalation. Kaplan–Meier curves illustrating the probability of not experiencing (A) relapses or (B) disease activity (as defined by relapses, gadolinium‐enhancing lesions, and new or enlarging T2 lesions) after de‐escalation to moderate‐efficacy therapy. Number of patients at risk and cumulative number of events at each year are reported below the graphs.

Patients remained on the initial ME DMT for a median of 0.8 years (IQR 0.3–2.5). At 2 years after de‐escalation, the cumulative risk was 38% (95% CI 31–44) for experiencing a relapse and 53% (95% CI 46–60) for experiencing disease activity (Figure 2). Three patients experienced EDSS increase during the follow‐up.

### 3.3 Factors Associated With Inflammatory Disease Activity Following De‐Escalation

Higher age was associated with a lower hazard rate of relapses (hazard ratio [HR] 0.96, 95% confidence interval [CI] 0.94–0.98; *p* < 0.001) and inflammatory disease activity (HR 0.96, 95% CI 0.94–0.98; *p* < 0.001) post de‐escalation. Inflammatory disease activity (relapses, gadolinium‐enhancing lesions, new or enlarging T2 lesions) during the last 6 months on HE DMT was associated with an increased hazard rate of post de‐escalation relapses (HR 1.60, 95% CI 1.04–2.48; *p* = 0.03) and disease activity (HR 2.05, 95% CI 1.45–2.91; *p* < 0.001). We did not find any associations between EDSS score increase on HE DMT, sex, disease duration, number of previous treatments, duration of HE DMT < 1 year, or baseline EDSS score, and subsequent relapses or inflammatory disease activity post de‐escalation (Supporting Information Table S1).

### 3.4 First Treatment Switch Following De‐Escalation

**FIGURE 3:**
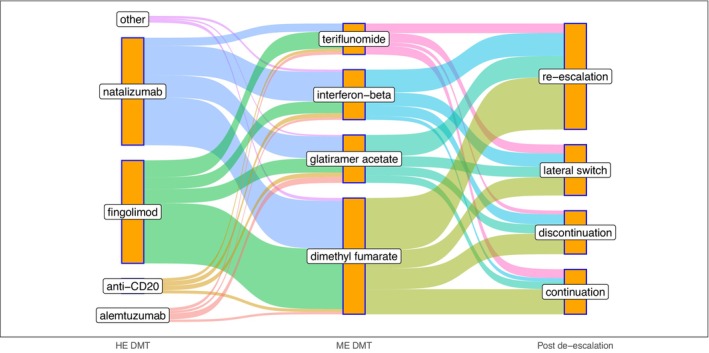
Treatment sequence for included patients. Sankey diagram visualizing the flow from HE DMTs before de‐escalation to ME DMTs as well as the first treatment switch after de‐escalation. HE DMT, high‐efficacy disease modifying therapy; ME DMT, moderate‐efficacy disease modifying therapy. Other HE DMTs included cladribine and daclizumab.

When analyzing the first treatment switch following de‐escalation, 144 (43.2%) re‐escalated to an HE DMT, 69 (20.7%) switched to another ME DMT, 61 (18.3%) continued the initial ME DMT, and 59 (17.7%) discontinued treatment entirely (Table 2, Figure 3). Patients who re‐escalated or switched to another ME DMT were younger at de‐escalation compared to those who continued the initial ME DMT with a mean age difference of −6.6 years (95% CI, −9.6 to −3.7; *p* < 0.001; *q* < 0.001) and −5.4 years (95% CI, −9.1 to −1.8; *p* = 0.004; *q* = 0.005), respectively. We did not find statistically significant differences in mean age at de‐escalation for the remaining pairwise comparisons (Supporting Information Table S2).

**TABLE 2:** Characteristics of the time during moderate‐efficacy therapy.

	Patients de‐escalating treatment (*n* = 333)
Follow‐up time, median (IQR), years	0.8 (0.3–2.5)
Total follow‐up time, years	629.6
Type of ME DMT, *n* (%)	
Dimethyl fumarate	158 (47.5)
Interferon‐beta and peginterferon	68 (20.4)
Glatiramer acetate	65 (19.5)
Teriflunomide	42 (12.6)
Reasons for discontinuation of ME DMT, *n* (%)^a^	
Adverse events	131 (48.2)
Disease activity^b^	93 (34.2)
Other reason	13 (4.8)
Pregnancy‐related	9 (3.3)
Progression	8 (2.9)
Patients' decision	5 (1.8)
Lack of patient compliance	4 (1.5)
Contra indication	3 (1.1)
Practical issues	< 3^c^
Anti‐drug antibodies	< 3^c^
Patient follow‐up was terminated	< 3^c^
Stable condition	< 3^c^
Missing	< 3^c^
Treatment strategy following de‐escalation, *n* (%)	
Re‐escalation	144 (43.2)
Lateral switch to other ME DMT	69 (20.7)
Continuation of ME DMT	61 (18.3)
Discontinuation of DMT	59 (17.7)
Age of patients following different treatment strategies post de‐escalation, mean (SD)^d^	
Re‐escalation	43.2 (8.9)
Lateral switch to other ME DMT	44.4 (10.6)
Continuation of ME DMT	49.9 (10.7)
Discontinuation of DMT	46.6 (10.1)

Abbreviations: DMT, disease‐modifying therapy; ME, moderate‐efficacy disease‐modifying therapy; SD, standard deviation.

^a^ Reasons for discontinuation as recorded by the treating physician.

^b^ Reason for stopping ME DMT reported as either due to disease activity, relapse, and/or MRI activity.

^c^ Masked to avoid individually identifiable information and to comply with the General Data Protection Regulation (GDPR).

^d^ Age at initiation of ME DMT.

Among the 144 patients that re‐escalated to an HE DMT, reasons for stopping the ME DMT before re‐escalating were relapse, MRI activity, or both in 85 (59.0%) patients and adverse events in 41 (28.5%) patients (Supporting Information Table S3).

## 4 Discussion

In this nationwide study with a complete follow‐up of the entire Danish DMT‐treated MS population, we included 333 individuals who underwent treatment de‐escalation for various reasons. Patients primarily de‐escalated from natalizumab and fingolimod to dimethyl fumarate. In the relatively short follow‐up, very few patients experienced an EDSS increase, while the cumulative risk of clinical or radiological disease activity within 2 years was 53%. We found that age is an important factor associated with a successful de‐escalation strategy, with older patients having a lower risk of inflammatory disease activity on ME DMT, and that patients who re‐escalated were younger at the initiation of ME DMT compared to those that continued ME DMT. Moreover, our study shows that disease activity shortly before de‐escalation is associated with an increased risk of inflammatory disease activity on ME DMT. When investigating the first treatment switch following de‐escalation, 43% reinitiated HE DMTs, primarily due to clinical or radiological breakthrough disease activity, whereas 39% of patients either stayed on the initial ME DMT or switched to another ME DMT before the end of follow‐up.

Some studies have previously addressed de‐escalation from natalizumab to ME DMTs [7, 14]. A clinical trial compared continuing natalizumab with either placebo or DMTs such as interferon beta 1A, glatiramer acetate, or methylprednisolone and showed that stopping natalizumab increased the risk of relapse or MRI activity compared to continuing natalizumab. This was supported by another clinical trial, albeit with a small sample size, that compared continuing natalizumab with de‐escalation from natalizumab to interferon beta 1b [20]. Further support comes from a small observational study that investigated glatiramer acetate after natalizumab with treatment discontinuation and found that both strategies were insufficient in controlling inflammation [21]. Another clinical trial investigating teriflunomide after natalizumab with no wash‐out period showed that 68% were free from any disease activity after 1 year, whereas 55% were free of new T2 lesions after 2 years [22, 23]. An MSBase observational study compared treatment with dimethyl fumarate, fingolimod, and ocrelizumab after natalizumab, showing comparable estimates of annualized relapse rates and hazard rates for dimethyl fumarate and fingolimod, but significantly lower estimates for ocrelizumab [24]. Taken together these studies indicate that ME DMTs apparently are not sufficient in controlling the pathogenic immune reactions that occur after stopping natalizumab. This fits with the overall results from our study, where 43.8% de‐escalated from natalizumab, and 53% experienced inflammatory disease activity within 2 years. Besides natalizumab, two recent studies have investigated de‐escalation from anti‐CD20 antibodies to cladribine and diroximel fumarate, respectively. Patients receiving cladribine showed stable serum biomarkers and no clinically evident disease activity 1 year after de‐escalation [25]. All patients receiving diroximel fumarate were stable for at least 1 year prior to de‐escalation and showed no signs of clinical or radiological disease activity 1 year after de‐escalation [26]. However, reactivation of disease activity is not very common after discontinuation of anti‐CD20 antibody therapy [27].

While a limited number of studies focus on de‐escalation, several discontinuation studies exist. Austrian investigators aimed to identify risk factors for relapses and disability progression in patients with relapsing MS who stopped IFN‐β or glatiramer acetate. They developed the VIADISCC score, which incorporated independent predictors of disease reactivation, namely age ≥ 45 years at discontinuation, MRI activity at discontinuation, and absence of relapses ≥ 4 years while on DMT [28]. Even though the study included a younger cohort and these patients discontinued DMT, our findings regarding age and disease activity prior to de‐escalation being associated with increased risk of inflammatory disease activity align well with the VIADISCC score findings. This is also in line with findings from a large meta‐analysis on discontinuing treatment, which suggests that the risk of relapse is significantly reduced, to less than 1%, after age 60 or with long‐term disease stability [29] and is also in line with another observational study conducted on retrospective data from several US clinics [30]. A randomized clinical trial (DISCOMS) investigated whether discontinuing therapy in patients 55 years and older with stable MS was inferior to continuing therapy. The study did not meet its primary outcome, reporting a higher proportion of patients with new inflammatory activity in those discontinuing DMT, primarily driven by one or two new MRI lesions. Post hoc analysis showed no difference in the proportion of patients with a new relapse, as well as no difference in EDSS worsening. In addition, a higher proportion of patients in the discontinuation group were satisfied with their treatment [31]. Recently, another randomized clinical trial (DOT‐MS) was suspended due to a higher proportion of participants with inflammatory disease activity in the group that discontinued compared to the group that continued first‐line DMTs. The DOT‐MS trial included participants 18 years and above without any inflammatory disease activity in the past 5 years. Compared to the DISCOMS trial, the DOT‐MS trial included younger patients with a shorter period of stable disease and reported a higher proportion of patients with inflammatory disease activity as well as a shorter time to experience inflammatory disease activity [32]. Also, a recent observational study investigating discontinuation of HE DMTs in patients 50 years and older found an increased rate of focal inflammatory activity in those discontinuing compared to those continuing HE DMT [33]. This indicates that discontinuing DMTs may increase the risk of new inflammatory activity, even in older patients. Furthermore, this prompts consideration of whether adopting a de‐escalation approach could be a more effective strategy.

One important limitation of this study is the lack of data on intentional de‐escalation due to stable disease. Because the data were collected in a real‐world setting, it includes patients stopping HE DMTs for various reasons and thereafter initiating an ME DMT. The most common causes of stopping the HE DMTs included adverse events, JC virus antibody positivity, disease activity, anti‐drug antibodies, and contraindications. Very few stopped HE DMT due to stable disease, but since deliberate de‐escalation is not a common treatment strategy in clinical practice, we did not expect to find many patients stopping HE DMTs for this reason. We included everyone de‐escalating from HE DMT to ME DMT, including patients with disease activity within 6 months before stopping their HE DMT (26.7%), and patients whose disease activity was recorded as the reason for stopping HE DMT (11.1%). We did this to provide a broad view of this treatment‐switching approach.

We also included induction therapies such as alemtuzumab and cladribine. These therapies with long‐lasting effects, might distort the evaluation of a de‐escalation strategy. However, we initially chose to include these induction therapies to reflect the broad real‐world treatment landscape, and, in the end, 22 patients de‐escalated from alemtuzumab and cladribine.

The mechanism of action of HE DMTs before de‐escalation can also affect patient outcomes. Due to the earlier availability of the anti‐trafficking DMTs, most patients in our study de‐escalated from natalizumab and fingolimod. We and others have previously reported clinical and radiological rebound activity after discontinuing fingolimod and natalizumab [34, 35, 36]. A recent study explored the implications of continuing versus discontinuing HE DMTs in patients older than 50 years with stable disease and found that discontinuing natalizumab and fingolimod was associated with a shorter time to relapse while discontinuing anti‐CD20 therapy was not [33]. We cannot exclude that the high proportion of patients in our study with disease reactivation can likely be due to rebound activity caused by discontinuation of fingolimod and natalizumab. Due to the limited follow‐up duration for patients starting anti‐CD20 therapies and the small sample size of the patient population undergoing de‐escalation within the DMSR, we cannot yet provide evidence on de‐escalation from anti‐CD20 therapy specifically.

The strength of our study lies in the quality of the data source, which provides evidence from a complete population treated with disease‐modifying therapies where data have been collected on regular clinical visits. By including the entire population who underwent de‐escalation for different reasons, we present the Danish experience with de‐escalation and the factors that could help to identify a population for which a de‐escalation strategy could be further studied.

In conclusion, in our cohort of 333 patients primarily de‐escalating from natalizumab and fingolimod for various reasons, the cumulative risk of inflammatory disease activity was 53% after 2 years. Younger age and disease activity before de‐escalation were associated with an increased risk of inflammatory disease activity after de‐escalation. These results suggest that in our cohort, de‐escalation was not sufficient to ensure disease stability. However, de‐escalation from other HE DMTs and in older patients with stable disease should be further explored.

## Author Contributions

**Frederik Elberling:** conceptualization, data curation, formal analysis, methodology, project administration, software, writing – review and editing. **Mie Reith Mahler:** formal analysis, project administration, writing – original draft, writing – review and editing. **Luigi Pontieri:** conceptualization, formal analysis, methodology, writing – review and editing. **Finn Sellebjerg:** conceptualization, writing – review and editing. **Melinda Magyari:** conceptualization, supervision, methodology, resources, writing – original draft, writing – review and editing.

## Disclosure

Frederik Elberling has received speaker honoraria from Roche and Sanofi.

Mie Reith Mahler has received support from Merck and Novartis for participation in scientific meetings and has received travel grants from the Danish Multiple Sclerosis Society.

Luigi Pontieri reports no disclosures relevant to the manuscript.

Finn Sellebjerg has served on scientific advisory boards for, served as consultant for, received support for congress participation, or received speaker honoraria from Biogen, Bristol Myers Squibb, Lundbeck, Merck, Novartis, Roche, and Sanofi Genzyme. His laboratory has received research support from Biogen, Merck, Novartis, Roche, and Sanofi Genzyme. He holds a professorship at the Faculty of Health Sciences and Medicine, University of Copenhagen sponsored by the Danish Multiple Sclerosis Society.

Melinda Magyari has served on scientific advisory boards for, as consultant for, received support for congress participation or speaker honoraria from Biogen, Sanofi, Roche, Novartis, Merck, and Moderna. Her group has received research support from Biogen, Genzyme, Roche, Merck, Novartis, and the Danish Multiple Sclerosis Society.

## Conflicts of Interest

The authors declare no conflicts of interest.

## Supporting information


Data S1.

## Data Availability

The data that support the findings of this study are available upon request and further approval from the Capital Region of Denmark and the board of the Danish Multiple Sclerosis Registry. The data are not publicly available due to privacy restrictions.

## Appendix A DMSG Study Group—Coinvestigators

**Table:**

Name	Location	Role	Contribution
Alex Heick, MD	Danish Multiple Sclerosis Center, Department of Neurology, Copenhagen University Hospital, Rigshospitalet Glostrup, Valdemar Hansens Vej 13, 2600 Glostrup Denmark	Site investigator	Data collection
Ana Isabel Figueira Jensen, MD	Danish Multiple Sclerosis Center, Department of Neurology, Copenhagen University Hospital, Rigshospitalet Glostrup, Valdemar Hansens Vej 132,600 Glostrup Denmark	Site investigator	Data collection
Jeppe Romme Christensen, MD, PhD	Danish Multiple Sclerosis Center, Department of Neurology, Copenhagen University Hospital, Rigshospitalet Glostrup, Valdemar Hansens Vej 132,600 Glostrup Denmark	Site investigator	Data collection
Jette Lautrup Battistini, MD, PhD, DMSci	Danish Multiple Sclerosis Center, Department of Neurology, Copenhagen University Hospital, Rigshospitalet Glostrup, Valdemar Hansens Vej 132,600 Glostrup Denmark Department of Clinical Medicine, aculty of Health and Medical Sciences, University of Copenhagen, Blegdamsvej 3B 2200 Copenhagen N Denmark	Site investigator	Data collection
Julie Richter Hansen, MD	Danish Multiple Sclerosis Center, Department of Neurology, Copenhagen University Hospital, Rigshospitalet Glostrup, Valdemar Hansens Vej 132,600 Glostrup Denmark	Site investigator	Data collection
Karen Ingrid Schreiber, MD, PhD	Danish Multiple Sclerosis Center, Department of Neurology, Copenhagen University Hospital, Rigshospitalet Glostrup, Valdemar Hansens Vej 13, 2600 Glostrup Denmark	Site investigator	Data collection
Morten Blinkenberg, MD, PhD	Danish Multiple Sclerosis Center, Department of Neurology, Copenhagen University Hospital, Rigshospitalet Glostrup, Valdemar Hansens Vej 132,600 Glostrup Denmark	Site investigator	Data collection
Per Soelberg Sørensen, MD, DMSci	Danish Multiple Sclerosis Center, Department of Neurology, Copenhagen University Hospital, Rigshospitalet Glostrup, Valdemar Hansens Vej 132,600 Glostrup Denmark	Site investigator	Data collection
Rikke Marie Jensen, MD	Danish Multiple Sclerosis Center, Department of Neurology, Copenhagen University Hospital, Rigshospitalet Glostrup, Valdemar Hansens Vej 13, 2600 Glostrup, Denmark	Site investigator	Data collection
Stephan Bramow, MD, PhD	Danish Multiple Sclerosis Center, Department of Neurology, Copenhagen University Hospital, Rigshospitalet Glostrup, Valdemar Hansens Vej 13, 2600 Glostrup, Denmark	Site investigator	Data collection
Viktoria Papp, MD, PhD	Danish Multiple Sclerosis Center, Department of Neurology, Copenhagen University Hospital, Rigshospitalet Glostrup, Valdemar Hansens Vej 13, 2600 Glostrup, Denmark	Site investigator	Data collection
Nils Koch‐Henriksen, MD, DMSci	The Danish Multiple Sclerosis Registry, Department of Neurology, Copenhagen University Hospital, Rigshospitalet Glostrup, Valdemar Hansens Vej 2, Entrance 8, 2nd floor, 2600 Glostrup, Denmark	Site investigator	Data collection
Morten Leif Munding Stilund, MD, PhD	Gødstrup Hospital, Department of Neurology, Physiotherapy and Occupational Therapy, Hospitalsparken 15, 7400 Herning, Denmark NIDO | Centre for Research and Education, Gødstrup Hospital, Hospitalsparken 25, 7400 Herning, Denmark	Site investigator	Data collection
Arkadiusz Weglewski, MD, PhD	Herlev and Gentofte Hospital, Department of Neurology, Herlev Ringvej 75, 2730 Herlev, Denmark Department of Clinical Medicine, Faculty of Health and Medical Sciences, University of Copenhagen, Blegdamsvej 3B, 2200 Copenhagen N, Denmark	Site investigator	Data collection
Henrik Kahr Mathiesen, MD, PhD	Herlev and Gentofte Hospital, Department of Neurology, Herlev Ringvej 75, 2730 Herlev, Denmark	Site investigator	Data collection
Henrik Boye Jensen, MD, PhD	Lillebælt Hospital, Department of Brain and Nerve Diseases, Sygehusvej 24, 6000 Kolding, Denmark Institute of Regional Health Research, Faculty of Health Sciences, University of Southern Denmark, Campusvej 55, 5230 Odense M, Denmark	Site investigator	Data collection
Mai Bang Poulsen MD, PhD	North Zealand Hospital, Copenhagen University Hospital, Department of Neurology, Dyrehavevej 29, 3400 Hillerød, Denmark	Site investigator	Data collection
Michael Langemark, MD	North Zealand Hospital, Copenhagen University Hospital, Department of Neurology, Dyrehavevej 29, 3400 Hillerød, Denmark	Site investigator	Data collection
Michael Broksgaard Jensen, MD, PhD	North Zealand Hospital, Copenhagen University Hospital, Department of Neurology, Dyrehavevej 29, 3400 Hillerød, Denmark	Site investigator	Data collection
Nadia Mubder Issa, MD	North Zealand Hospital, Copenhagen University Hospital, Department of Neurology, Dyrehavevej 29, 3400 Hillerød, Denmark	Site investigator	Data collection
Ásta Milthers Theódórsdóttir, MD, PhD	Odense University Hospital, Department of Neurology, J. B. Winsløws Vej 4, 5000 Odense, Denmark	Site investigator	Data collection
Helle Hvilsted Nielsen, MD, PhD	Odense University Hospital, Department of Neurology, J. B. Winsløws Vej 4, 5000 Odense, Denmark Department of Clinical Research, University of Southern Denmark, J. B. Winsløws Vej 4, 5000 Odense, Denmark	Site investigator	Data collection
Manuel Demirovic, MD	Odense University Hospital, Department of Neurology, J. B. Winsløws Vej 4, 5000 Odense, Denmark	Site investigator	Data collection
Zsolt Laszlo Illés, MD, PhD, DMSci	Odense University Hospital, Department of Neurology, J. B. Winsløws Vej 4, 5000 Odense, Denmark Department of Clinical Research, University of Southern Denmark, J. B. Winsløws Vej 4, 5000 Odense, Denmark	Site investigator	Data collection
Homayoun Roshanisefat, MD, PhD	Department of Neurology, Slagelse Hospital, Fælledvej 11, 4200 Slagelse	Site investigator	Data collection
Masoud Falah, MD	Department of Neurology, Slagelse Hospital, Fælledvej 11, 4200 Slagelse	Site investigator	Data collection
Nasrin Asgari, MD, PhD, DMSci	Department of Neurology, Slagelse Hospital, Fælledvej 11, 4200 Slagelse Department of Clinical Research, University of Southern Denmark, J. B. Winsløws Vej 4, 5000 Odense, Denmark	Site investigator	Data collection
Afzal Gardezi, MD	Hospital of Southern Jutland, Department of Neurology, University of Southern Denmark, Kresten Philipsens Vej 15, 6200 Aabenraa, Denmark	Site investigator	Data collection
Matthias Kant, MD	Hospital of Southern Jutland, Department of Neurology, University of Southern Denmark, Kresten Philipsens Vej 15, 6200 Aabenraa, Denmark	Site investigator	Data collection
Thor Petersen, MD, PhD	Hospital of Southern Jutland, Department of Neurology, University of Southern Denmark, Kresten Philipsens Vej 15, 6200 Aabenraa, Denmark	Site investigator	Data collection
Tobias Sejbæk, MD, PhD	Southwest Jutland Hospital, Department of Neurology, University Hospital of Southern Denmark, 6700 Esbjerg, Denmark Institute of Regional Health Research, Faculty of Health Sciences, University of Southern Denmark, Campusvej 55, 5230 Odense M, Denmark	Site investigator	Data collection
Claudia Christensen, MD, PhD	Aalborg University Hospital, Department of Neurology, Reberbanegade 15, 9100 Aalborg, Denmark	Site investigator	Data collection
Inga Urbonaviciute, MD	Aalborg University Hospital, Department of Neurology, Reberbansgade 15, 9100 Aalborg, Denmark	Site investigator	Data collection
Ismael Barzinji, MD	Aalborg University Hospital, Department of Neurology, Reberbanegade 15, 9100 Aalborg, Denmark	Site investigator	Data collection
Jakob Schäfer, MD	Aalborg University Hospital, Department of Neurology, Reberbanegade 15, 9100 Aalborg, Denmark	Site investigator	Data collection
Camilla Mærsk‐Møller, PhD	Aarhus University Hospital, Department of Neurology, PJJ Boulevard 165, J504, 8200 Aarhus N, Denmark	Site investigator	Data collection
Caroline Winther Tørring, MD, PhD	Aarhus University Hospital, Department of Neurology, PJJ Boulevard 165, J504, 8200 Aarhus N, Denmark	Site investigator	Data collection
Kristina Bacher Svendsen, MD, PhD	Aarhus University Hospital, Department of Neurology, PJJ Boulevard 165, J504, 8200 Aarhus N, Denmark	Site investigator	Data collection
Peter Vestergaard Rasmussen, MD, PhD	Aarhus University Hospital, Department of Neurology, PJJ Boulevard 165, J504, 8200 Aarhus N, Denmark	Site investigator	Data collection
Georgi P. Sirakov, MD	Regionshospital Viborg, Hospitalsenhed Midt, Department of Neurology, Heibergs Allé 4, 8800 Viborg	Site investigator	Data collection
Sivagini Prakash, MD	Regionshospital Viborg, Hospitalsenhed Midt, Department of Neurology, Heibergs Allé 4, 8800 Viborg	Site investigator	Data collection
Lars Kristian Storr, MD, PhD	Zealand University Hospital, Department of Neurology, Sygehusvej 10, 4000 Roskilde, Denmark	Site investigator	Data collection
Lena Christina Roug, MD	Zealand University Hospital, Department of Neurology, Sygehusvej 10, 4000 Roskilde, Denmark	Site investigator	Data collection
Monika Katarzyna Góra, MD	Zealand University Hospital, Department of Neurology, Sygehusvej 10, 4000 Roskilde, Denmark	Site investigator	Data collection
Said Ashna, MD	Zealand University Hospital, Department of Neurology, Sygehusvej 10, 4000 Roskilde, Denmark	Site investigator	Data collection
Anja Thormann, MD, PhD	Danish Multiple Sclerosis Center, Department of Neurology, Copenhagen University Hospital, Rigshospitalet Glostrup, Valdemar Hansens Vej 13, 2600 Glostrup, Denmark	Site investigator	Data collection
Thor Chalmer, MD, PhD	Danish Multiple Sclerosis Center, Department of Neurology, Copenhagen University Hospital, Rigshospitalet Glostrup, Valdemar Hansens Vej 13, 2600 Glostrup, Denmark	Site investigator	Data collection
Jesper Nørregaard, MD	Danish Multiple Sclerosis Center, Department of Neurology, Copenhagen University Hospital, Rigshospitalet Glostrup, Valdemar Hansens Vej 13, 2600 Glostrup, Denmark	Site investigator	Data collection
Helene Højsgaard Chow, MD, PhD	Danish Multiple Sclerosis Center, Department of Neurology, Copenhagen University Hospital, Rigshospitalet Glostrup, Valdemar Hansens Vej 13, 2600 Glostrup, Denmark	Site investigator	Data collection
