# Supplementary material for: De‐Escalation of Disease‐Modifying Therapy in Multiple Sclerosis—A Danish Nationwide Cohort Study
Source: Eur J Neurol. 2025 Feb 2;32(2):e70042. doi: 10.1111/ene.70042 (PMC11788534; doi:10.1111/ene.70042)
Supplement: Supplementary file 1 — Data S1. [file ENE-32-e70042-s001.docx]

# Supplemental Material

*Table 1 – Hazard ratios of time to relapse and inflammatory disease activity derived from Cox models.*

|  | **Relapses** | | **Disease activity** | |
| --- | --- | --- | --- | --- |
|  | **Hazard ratio (95% CI)** | **P-value** | **Hazard ratio (95% CI)** | **P-value** |
| **EDSS score increase on HE DMT** | 1.49 (0.79 to 2.83) | 0.22 | 1.41 (0.82 to 2.45) | 0.22 |
| **Disease activity on HE DMT** | 1.60 (1.04 to 2.48) | 0.03 | 2.05 (1.45 to 2.91) | <0.001 |
| **Male Sex** | 0.64 (0.40 to 1.02) | 0.06 | 0.78 (0.54 to 1.13) | 0.19 |
| **Age** | 0.96 (0.94 to 0.98) | <0.001 | 0.96 (0.94 to 0.98) | <0.001 |
| **Disease duration** | 1.01 (0.97 to 1.04) | 0.65 | 1.00 (0.97 to 1.03) | 0.83 |
| **Number of previous treatments** | 1.01 (0.84 to 1.21) | 0.95 | 1.10 (0.96 to 1.27) | 0.17 |
| **Duration of HE DMT < 1 year** | 0.92 (0.55 to 1.53) | 0.75 | 0.89 (0.58 to 1.36) | 0.59 |
| **EDSS score before ME DMT** | 1.02 (0.92 to 1.14) | 0.67 | 1.00 (0.91 to 1.10) | 0.99 |

HE: high-efficacy disease modifying therapy. EDSS: Expanded Disability Status Scale. CI: confidence interval.

*Table 2 – Pairwise comparisons of mean age at initiation of ME DMT among patients who either re-escalate, lateral switch to another ME DMT, continue ME DMT or discontinue DMT.*

| **Comparison** | **Mean age difference, years** | **95% confidence interval** | **p-value** | **FDR adjusted p-value (q-value)** |
| --- | --- | --- | --- | --- |
| **Re-escalation vs. lateral switch** | -1.2 | -4.2 to 1.7 | 0.41 | 0.39 |
| **Re-escalation vs. continuation** | -6.6 | -9.6 to -3.7 | < 0.001 | < 0.001 |
| **Re-escalation vs. discontinuation** | -3.4 | -6.4 to -0.4 | 0.03 | 0.05 |
| **Lateral switch vs. continuation** | -5.4 | -9.1 to -1.8 | 0.004 | 0.005 |
| **Lateral switch vs. discontinuation** | -2.2 | -5.8 to 1.4 | 0.23 | 0.24 |
| **Continuation vs. discontinuation** | 3.2 | -0.5 to 6.9 | 0.09 | 0.11 |

ME: moderate efficacy; DMT: disease-modifying therapy; FDR: false discovery rate.

*Table 3 – Reasons for discontinuation of ME DMT before re-escalation*

| **Reason** | **n (%)** |
| --- | --- |
| Disease activity | 85 (59.0) |
| Adverse events | 41 (28.5) |
| Other reason | 4 (2.8) |
| Progression | 4 (2.8) |
| Pregnancy-related | 4 (2.8) |
| Patient’s decision | 3 (2.1) |
| Practical issues | < 3 ^a^ |
| Antibodies | < 3 ^a^ |

ME DMT: moderate efficacy disease modifying therapy
^a^ Masked to avoid individually identifiable information and to comply with the General Data Protection Regulation (GDPR).
